# Temporal variations in turbidity in an oil sands pit lake

**DOI:** 10.1007/s10652-018-9632-6

**Published:** 2018-11-10

**Authors:** Edmund Tedford, Geoff Halferdahl, Roger Pieters, Gregory A. Lawrence

**Affiliations:** 1 Environmental Fluid Mechanics Group, Department of Civil Engineering, University of British Columbia, Vancouver, BC V6T 1W5 Canada; 2 Syncrude Canada Ltd., 9421 – 17 Avenue NW, Edmonton, AB T6N 1H4 Canada

**Keywords:** Turbidity, Stratification, Mixing, Pit lake, Oil sands mine

## Abstract

We investigated temporal variations in turbidity in Base Mine Lake, an oil sands pit lake, located in northeast Alberta, Canada. The lake has a surface area of 7.8 km^2^, is 9 m deep, and exhibits seasonal thermal stratification similar to that of natural dimictic lakes. Water turbidity was measured continuously throughout the year with moored sensors, supplemented with turbidity profiles and bottle samples, from sites on the lake. During summer there was a gradual exponential (e-folding time of 25 days) decrease in epilimnetic turbidity due to relatively steady settling of solids from the epilimnion through the thermocline to the hypolimnion. Rapid oscillations (periods of approximately 1 day) in turbidity during summer were due to wind driven internal waves. Convective cooling and wind-shear driven stirring during fall storm events increased the turbidity to an annual high just before ice-on. During these storm events, similarity scaling indicated wind shear imparted greater energy than convective cooling to the turbulence at the base of the water column. Ice suppressed wind forcing and resulted in a rapid decrease in turbidity. The rate of decrease in turbidity following ice-on was initially greater than the rate of decrease in the epilimnion during summer, and then slowed until the under-ice turbidity was a relatively constant value which was sustained during the latter half of the ice-on period. The minimum turbidity during winter was greater than the minimum during summer. Following ice-melt in spring, wind driven stirring increased turbidity until summer stratification began.

## Introduction

Turbidity is an important characteristic of aquatic ecosystems. It controls the penetration of sunlight into the water column, thereby affecting the absorption of solar energy and the resulting distribution of heat [7, 8], the level of biological activity [6], and the aesthetic appearance of the water body [19]. Many factors affect turbidity in both natural and man-made lakes over a wide range of time scales.

Dissolved organic matter (e.g. humic acids), suspended organic particles (e.g. phytoplankton), and suspended inorganic particles (e.g. clastic materials, [1, 23] can all contribute to turbidity. They may originate from a variety of sources, both allochthonous (such as tributary inputs), and autochthonous (such as biological production, shoreline erosion, and re-suspension from the bottom). For example, turbidity can be controlled by the tributary inflow of inorganic particles such as glacial fines that are slow to settle [18, 24]. In shallow water bodies, including lakes [3] and tailings ponds [14], high turbidity can result from re-suspension of bottom sediments by wind driven currents.

**Fig. 1:**
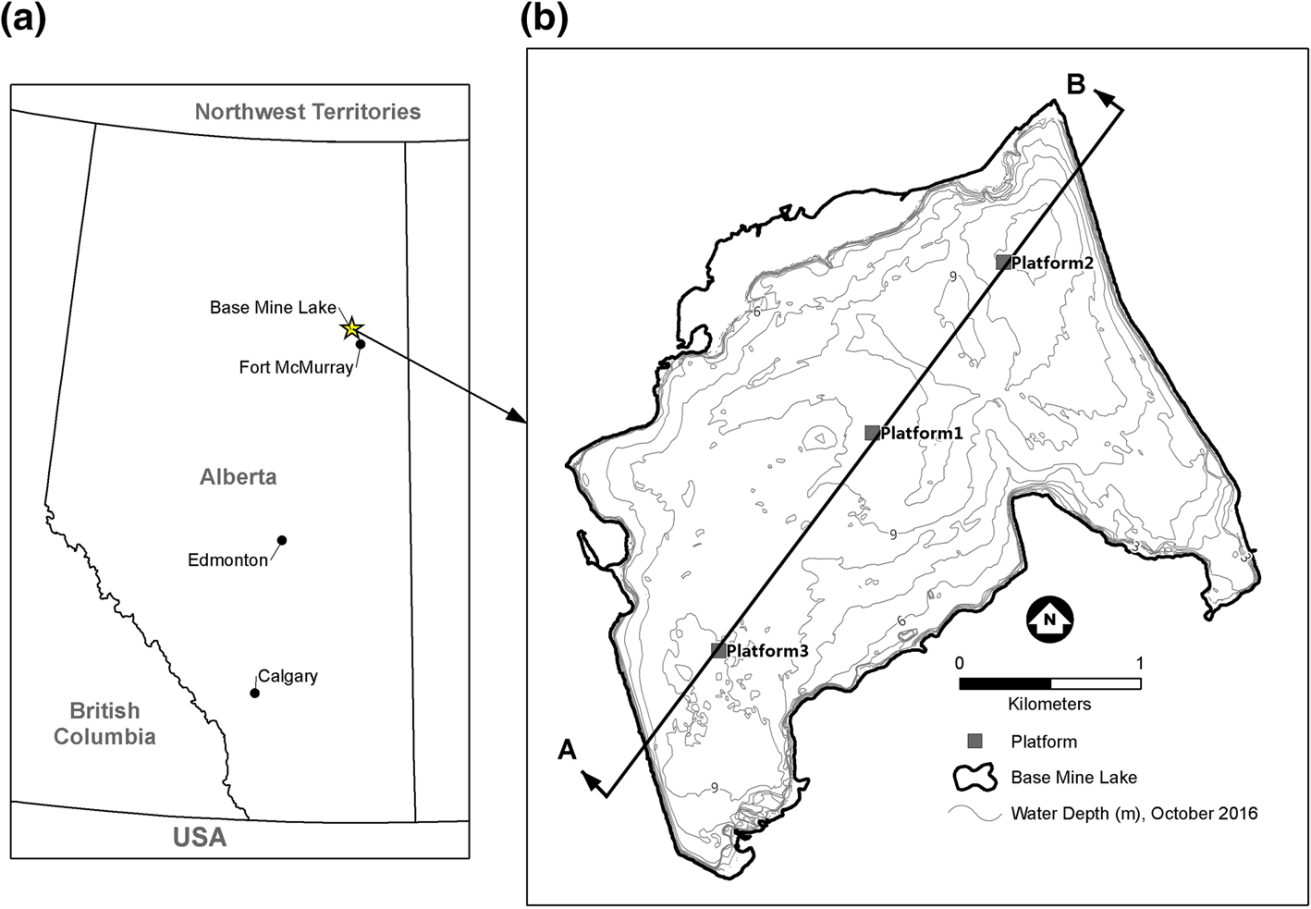
Base Mine Lake location (starred) north of Fort McMurray, Alberta, Canada at 57°1′N, 111°37′W, 308 m above sea level. The insert (**b**) shows the locations of the three platforms overlain on the October 2016 water depths (m). The line through the platforms shows the location of the cross-section in Fig. 2

**Fig. 2:**
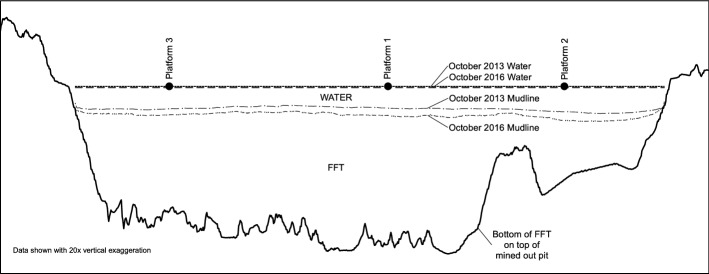
Base Mine Lake 20 × vertical exaggeration cross-section looking NE (location shown in Fig. 1). The horizontal shore to shore distance is approximately 4.1 km; the maximum FFT thickness is approximately 45 m and the average water depth was approximately 9 m in October 2016. Between October, 2013 and October, 2016 the water level rose due to freshwater inflows while the mudline (water-FFT) interface) fell due to settling (see Sect. 2 for details). The water surface and mudline for these two dates are included

Here we examine turbidity in Base Mine Lake (BML) a unique man-made water body in the Athabasca oil sands region in Alberta, Canada (Fig. 1). The lake is the first full scale demonstration end pit lake in the region and consists of water with an average depth of 9 m, covering a layer of fluid fine tailings (FFT) of up to 45 m deep (Fig. 2). BML has low biological activity (low primary production due to limited light penetration), and small inflows that have much lower turbidity (< 10 NTU) than the lake water (> 100 NTU). Therefore, Lawrence et al. [12] hypothesize that BML turbidity is governed by the internal cycling of fine inorganic particles, and that during settling, these particles form a layer of increased turbidity at the base of the water cap, similar to the fluid mud often found in estuaries [15]. This layer is far less dense and more mobile than the underlying gel-like FFT, and is subsequently re-suspended by hydrodynamic processes that are not strong enough to erode the underlying FFT. The goal of the present paper is to investigate these hydrodynamic processes, the time scales over which they act, and the resulting temporal variations in turbidity observed in BML from May 2013 until August 2016. The study site and data collection are described in Sects. 2 and 3 respectively. Results are given in Sect. 4. Discussion and conclusions are given in Sects. 5 and 6 respectively.

## Site description

Base Mine Lake (57°1′N, 111°37′W, elevation 308 m, surface area 7.8 km^2^) is located in a mined out oil sands pit at the Syncrude Canada Ltd. base mine site in northeast Alberta, Canada (Fig. 1). Until late 2012 BML was an active tailings pond storing large quantities of FFT overlain by process-affected water. In November 2012 BML was disconnected from tailings operations, and fresh water was added to increase the water depth sufficient to prevent wind-wave induced erosion of the FFT that forms much of the lake bottom as shown in Fig. 2 [11]. The sides and bottom of the pit are a combination of undisturbed stratigraphy, overburden dumps and engineered dams made of compacted overburden, see Fig. 2.

During the study period, from May 2013 to August 2016, the average depth of the water increased from approximately 5.2–8.9 m. Of this 3.7 m increase, approximately 2.3 m was due to the on-going settling of FFT, and 1.4 m was due to fresh water (turbidity < 10 NTU) piped into the lake from a nearby reservoir. The net inflow from surface runoff plus precipitation onto the lake surface minus evaporation from it was negligible. The volume of the water increased from approximately 35 × 10^6^ – 62 × 10^6^ m^3^ as the volume of the FFT decreased approximately from 184 × 10^6^ to 167 × 10^6^ m^3^ over the study period. As a consequence of the fresh water inputs, the concentration of total dissolved solids in the lake water decreased from approximately 2.3 g/L in May 2013 to approximately 1.6 g/L in August 2016.

## Data collection

### Meteorological measurements

A meteorological station was mounted on a floating platform anchored in the center of the lake (Platform 1 or P1, Fig. 1). Incoming and outgoing long- and short-wave radiation were measured from a boom above the water surface; and relative humidity and air temperature were measured with shielded sensors. Wind speed and direction were measured with an anemometer 3.3 m above the lake surface. A secondary meteorological station located 1.5 km northeast of the lake was used to fill gaps in the wind data.

### Temperature chains and moored turbidity sensors

Time-series measurements of temperature were obtained initially at the central platform (P1, 10 depths, February 2014 to May 2015) and then at both the northeast platform (P2, 11 depths) and southwest platform (P3, 10 depths) using self-contained temperature loggers (RBR SoloT, 0.002 °C accuracy) on moorings. Each of these temperature chains included two pressure loggers to verify the vertical stability of the mooring. In May of 2015 two self-contained turbidity loggers (RBRDuo with Seapoint turbidity sensor) were added to the temperature chain at the southwest platform, P3, at depths of 2.5 m and 7.5 m. The temperature and pressure loggers sampled every 10 s, and the turbidity loggers sampled every 30 s. During May, June and July of 2015 an RBRConcerto was used at P3 in place of RBRSoloTs. This instrument had an accuracy of approximately 0.25 °C.

### Profiling data (turbidity and temperature) and bottle samples

In the first year of the study, monthly from May to October 2013 and in February 2014, turbidity and total suspended solids (TSS) data were obtained from bottle samples collected throughout the water depth at various locations. The samples were analyzed in the laboratory with a calibrated bench top turbidity meter. Coincident water temperature data were collected in the field with a YSI Castaway (accuracy 0.01 °C). We will focus on the turbidity measurements in the present paper, because of its relative simplicity (including frequent sensor calibration checks) compared to the sampling and laboratory work associated with determining TSS, and because it is a more direct measure of water clarity (and light penetration). Preliminary analyses (not shown) indicate the turbidity in NTU is approximately twice the TSS in mg/L.

Monthly from May to October 2014, 2015 and 2016, and in March 2015, turbidity and temperature profiles were obtained with a Seabird 19plus (accuracy 0.0005 °C) equipped with a Seapoint turbidity sensor at multiple locations.

## Results

**Fig. 3:**
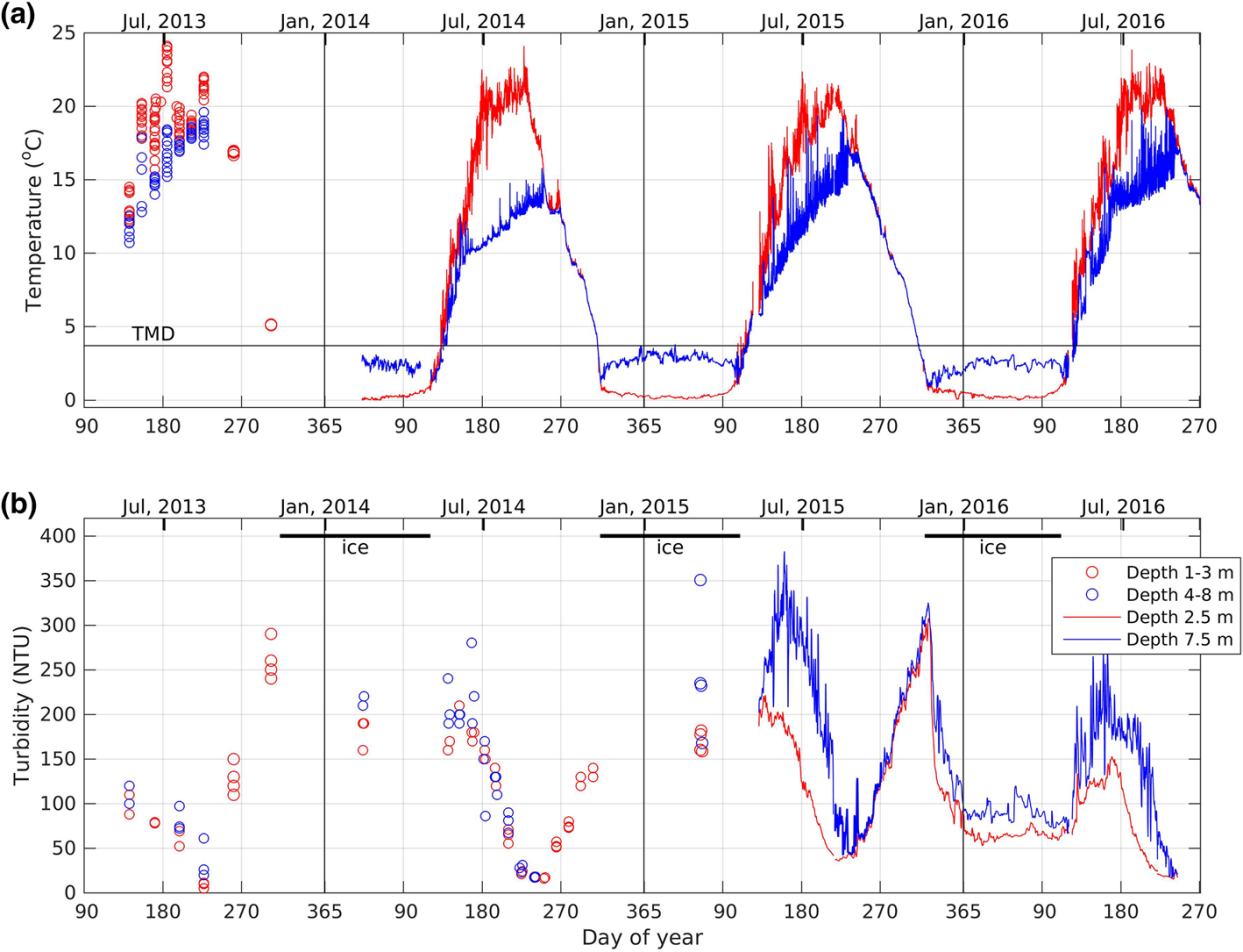
Time series of **a** temperature and **b** turbidity in Base Mine Lake from May 2013 to August 2016 (daily moving average)

**Fig. 4:**
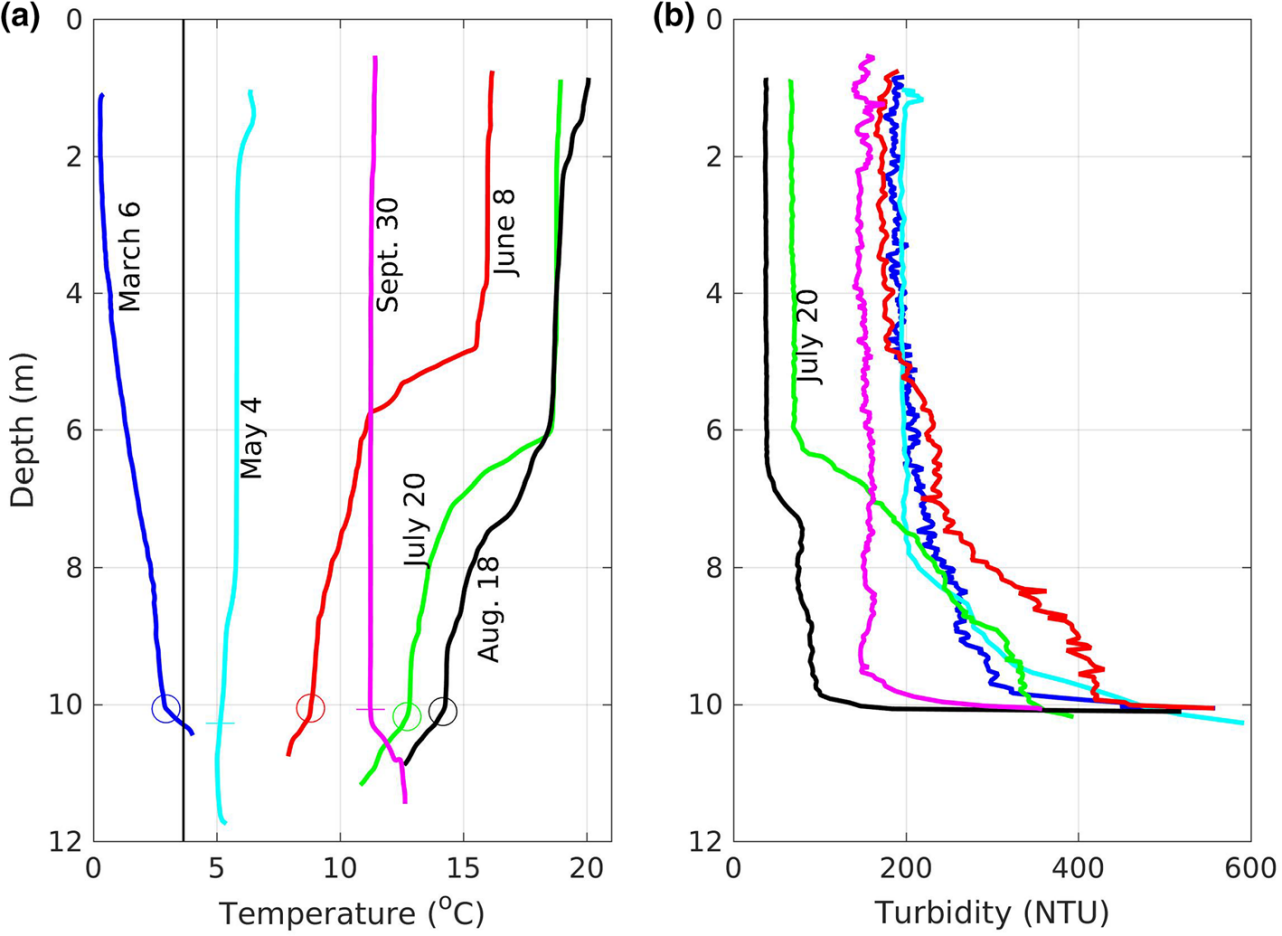
Vertical profiles of **a** turbidity and **b** temperature collected during 2015 at the northeast platform (P2). The depth at which the turbidity exceeds the threshold of the sensor is marked by a circle in the temperature profiles. **a** The vertical line marks the temperature of maximum density

Base Mine Lake exhibits dimictic behaviour, mixing throughout the water column in May (immediately following ice off), and in September and October, following the breakdown of summer thermal stratification and before ice-on (Fig. 3a). Over the 40 month study period, the largest variations of turbidity coincided with the seasonal cycle in mixing: increasing during spring turnover, decreasing during summer stratification, increasing again during fall turnover, and decreasing again after ice formation (Fig. 3b). This cycle in turbidity became clearer as bottle samples in 2013 were augmented with periodic turbidity profiles in 2014, and further augmented with moored turbidity loggers in 2015 and 2016. The data from the moored turbidity loggers also revealed variations at shorter time scales from the annual cycle, providing evidence of the influence of wind driven motions and thermal convection. In this section we focus on 2015, and describe the seasonal and shorter time scale variations in turbidity and thermal stratification using both time series (Fig. 3) and selected vertical profiles (Fig. 4). We will also briefly discuss interannual variability in turbidity.

*The timing of turnover and stratification*

The timing of ice cover and lake turnover varies from year to year (see Table 1) and we define the “seasons” accordingly. We define the period between ice-on and ice-off as winter, between ice-off and the onset of summer stratification as spring, between the onset of summer stratification and the onset of fall turnover as summer, and between the onset of fall turnover and ice-on as fall.

**Table 1:** Summary of seasonal turbidity (NTU) extremes at shallow depths (1–3 m) and the timing of ice-on, ice-off, and the onset of summer stratification and fall turnover. Note that the exact timing of these events is often uncertain, our best estimates are given here

Year	2013	2014	2015	2016
Winter min. (NTU)	–	*160*	*159*	53
Ice-off	–	May 1	Late April	April 27
Spring max. (NTU)	*99* ^a^	*177*	221	153
Stratification onset	Late May^b^	May 30	June 9	June 23
Summer min. (NTU)	*5*	*10*	36	16
Fall turnover	Early Sept.	Sept. 7	August 28	August 27
Fall max. (NTU)	*260*	*138*	308	–
Ice-on	Nov 10	Nov 11	Nov 20	

^a^Italics mark turbidity measured from bottle samples before the continuous moored turbidity loggers were installed

^b^Estimate only

### Winter

Our time series data show that prior to ice-on in mid-November the water is homogeneous and cooler than the temperature of maximum density (see Fig. 3a). Once the lake is fully ice-covered the upper portion of the water column cools approaching 0 °C, while the deeper water warms approaching the temperature of maximum density (~ 3.7 °C). The turbidity decreases rapidly at ice-on and then asymptotes to a relatively constant level that is maintained until ice-off (Fig. 3b).

The profiles of temperature and turbidity collected under ice (6 March 2015, day 65, Fig. 4) corroborate the time series data. They show that the water temperature increases from close to 0 °C immediately under the ice, to the temperature of maximum density near the bottom of the water cap (~ 10.5 m). This reverse stratification is similar to conditions under ice in natural lakes and occurs every winter in BML (Fig. 3a). The turbidity increased with depth from 200 NTU in the upper 5 m of the water column to 330 NTU at a depth of 9.8 m (Fig. 4b). Below a depth of 9.8 m the turbidity increased dramatically indicating the presence of a fluid mud layer. The turbidity exceeded the threshold of the sensor (about 800 NTU) at approximately 10 m depth. Note, unlike summer, the turbidity in winter displays persistent horizontal gradients between the south and north end of the lake (not shown).

### Spring and summer

The first profiles collected after ice-off (4 May 2015, day 124) show both a relatively uniform turbidity of 200 NTU (Fig. 4b), and a uniform temperature of 6 °C (Fig. 4a) in the top 8 m. Between 8 and 10 m depth, the turbidity increased to 500 NTU, while the temperature decreased to 5 °C. Summer thermal stratification developed soon after these profiles were collected.

Like other temperate and northern lakes, BML stratifies every summer (for example, from approximately 9 June–28 August 2015, day 160–240, Fig. 3a). When the moored turbidity sensors were first deployed (11 May 2015, day 131) the temperature and turbidity were essentially the same at 2.5 m and 7.5 m (Fig. 3b). As stratification developed in both 2015 and 2016 the turbidity remained elevated at a depth of 2.5 m depth and increased at 7.5 m depth. Once the thermal stratification was well established the turbidity began to decline first at a depth of 2.5 m and then at a depth of 7.5 m (between 9 June and 19 July 2015, day 160–200, Fig. 3b). This decline in turbidity continued until the breakdown of thermal stratification, and occurred in all 4 years of data collection.

The vertical profiles in Fig. 4 are consistent with the time series data. Between 4 May and 8 June 2015 (day 124–159) the lake became temperature stratified, while the turbidity decreased slightly in the epilimnion and increased below the epilimnion. Between 8 June and 20 July 2015 (day 159–201) the epilimnion warmed approximately 3 °C and the hypolimnion warmed by approximately 4 °C, while the thermocline shifted downward by approximately one meter. During this period the turbidity declined particularly in the upper layer. Between 20 July and 18 August 2015 (day 201–230) the lower layer warmed by 1–2 °C, while the turbidity continued to decline at all depths. The overall decline in turbidity is primarily due to particle setting and will be discussed in Sect. 4.2.1.

It should be noted that throughout summer, density stratification supports wind driven internal seiching resulting in high frequency variations in temperature and turbidity (Fig. 3), which will be described in greater detail in Sect. 4.2.2.

#### Particle settling from the epilimnion

**Fig. 5:**
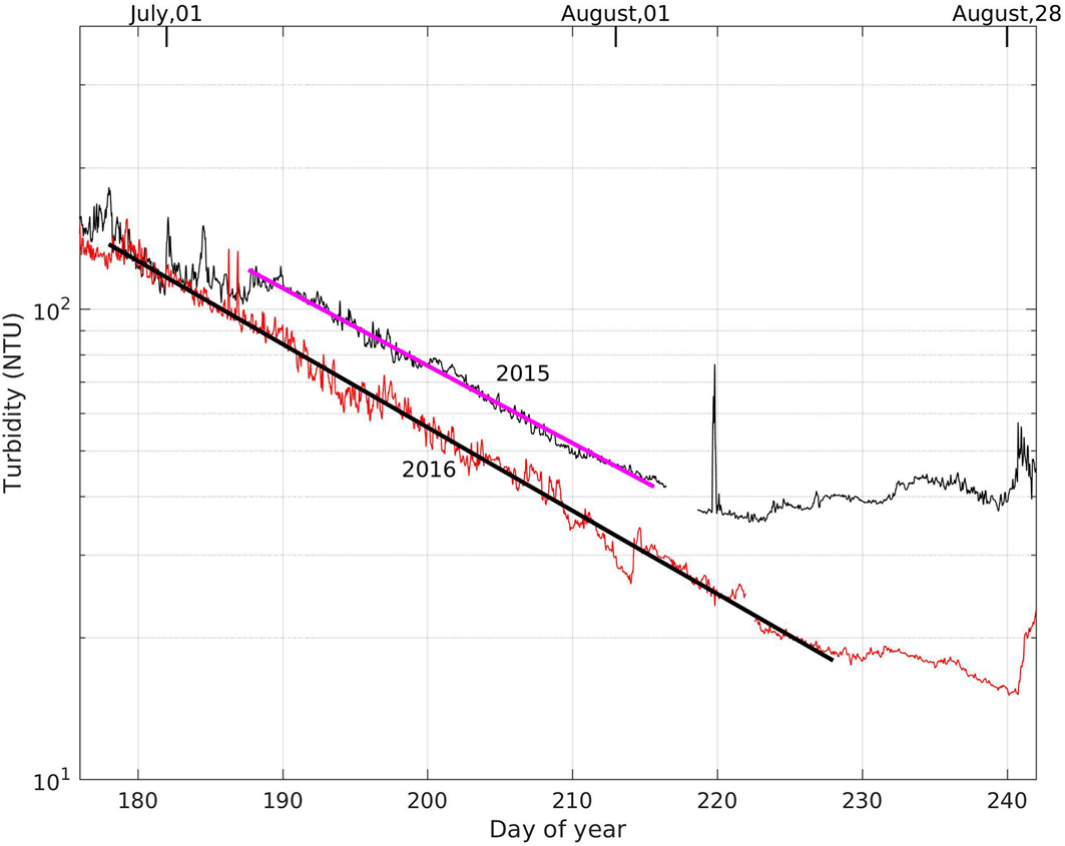
Summer turbidity (hourly average) in 2015 and 2016. The thick black and magenta lines represent log linear least square fits for the associated range of time. The e-folding times are 26 days in 2015 and 24 days in 2016

During summer stratification, the turbidity at 2.5 m decreases exponentially; see Fig. 5. For example: between 7 July and 3 August 2015 (day 188–215) the turbidity decreases with an e-folding time of 26 days (4.1% decrease per day). For 2016, the corresponding e-folding time is 24 days (3.8% decrease per day).

Here we apply the approach of Smith [20] and Reynolds [17] to explain the observed exponential decrease in turbidity and provide an estimate of the effective particle settling velocity. We make the following assumptions:

1. Under still water conditions, small (< 100 microns) spherical particles fall through the water column at the Stokes settling velocity:
  1 $$ V_{S} = \frac{{g{'} D^{2} }}{18 \nu } $$
  where $g' = g\left( {\rho_{p} - \rho_{w} } \right)/\rho_{w}$, and $\rho_{p}$ and $\rho_{w}$ are the densities of the particles and water, respectively; *D* the diameter of the particles; and $\nu$ the kinematic viscosity of water. We assume the density of the particles is 2650 kg m^−3^.
2. The epilimnion is subject to daily vertical mixing so that the particle concentration, like turbidity, is considered uniform. The total mass of particles in the epilimnion is $\rho_{p} C\forall$, where $C$ is the concentration of particles in the epilimnion and $\forall$ is the volume of the epilimnion. Smith [20] shows that, if the period between mixing events is small compared to the e-folding time (the time required for the concentration to decrease by 1/e), then this assumption is valid. This assumption is satisfied since epilimnetic mixing in BML, due to either wind or thermal convection, generally occurs on a daily basis, while the e-folding time in BML is almost a month.
3. Particles are falling from the epilimnion into the hypolimnion with velocity, $V_{S}$, and once they enter the hypolimnion they remain there. Thus the flux of particles leaving the epilimnion is $\rho_{p} CV_{S} A$, where $C$ is the mass concentration of particles in the epilimnion, and $A$ is the surface area of the interface between the epilimnion and hypolimnion.
4. The average depth of the epilimnion, $h = \forall /A$, remains constant. Note that towards the end of summer this assumption is violated, because the epilimnion deepens rapidly.
5. The fresh water inflow during summer does not affect the turbidity of the epilimnion. The maximum fresh water inflow is 1.8 × 10^6^ m^3^/month (see [5], Fig. 3), which for a 5 m deep epilimnion corresponds to a maximum turbidity decrease of 0.03%/day, much less than the observed decrease.

Equating the rate of change of particle mass in the epilimnion with the flux of particles settling into the hypolimnion gives:

2 $$ \frac{{d(\rho_{p} CAh)}}{dt} = - \rho_{p} CV_{S} A $$

and after integration:

3 $$ C(t) = C_{o} \left( t \right)\,{ \exp }\left\{ { - \frac{t}{\tau }} \right\} $$

where the e-folding time scale:

4 $$ \tau = \frac{h}{{V_{S} }} $$

The e-folding time scale equals the time it would take for a particle to settle through the epilimnion. Typically the epilimnion in Base Mine Lake has a depth of about 5 m so that in both 2015 and 2016, $V_{S} \approx 0.2$ m/day. Rearranging the above equations gives an effective settling diameter of:

5 $$ D = \sqrt {\frac{18 \nu h}{{g{'} \tau }}} $$

setting $v = 10^{ - 6} \;{\text{m}}^{2} \;{\text{s}}^{ - 1} ,\; h = 5 \;{\text{m}},\;g^{'} = 16.2\; {\text{m}}\;{\text{s}}^{ - 2} \; {\text{and}}\; \tau = 2.16 \times 10^{6} \; {\text{s }}\left( {25 \;{\text{days}}} \right)$, yields $D = 1.6 \times 10^{ - 6} {\text{m}}$. This value is an estimate of the effective diameter of spherical particles that would result in an e-folding time of 25 days. In reality the particles are not of uniform size, nor are they spherical; nevertheless, the calculated value is consistent with median particle size of samples taken from Base Mine Lake FFT (G. Halferdahl, personal communication).

#### Wind-driven turbidity variations

Every summer, wind events drive tilting, upwelling and oscillations of the thermocline in lakes, including Base Mine Lake. Here we examine one of these events from the summer of 2015 and the connection between motion of the thermocline and variations in turbidity.

**Fig. 6:**
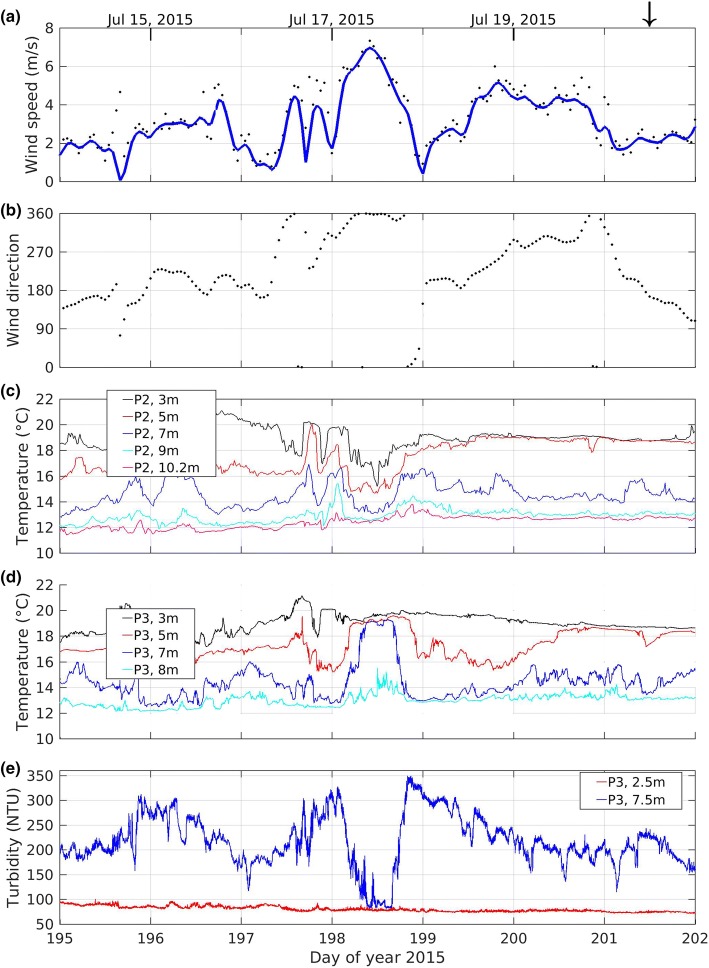
**a** Wind speed (hourly), **b** wind direction (hourly), **c**, **d** water temperature (10 s) at the northeast and southwest platforms respectively, and **e** turbidity (30 s) at the southwest platform. The downward arrow marks the time of the July 20 profiles in Fig. 4

We focus on the variations that were excited by a strong wind (> 6 m/s and sustained for more than 6 h) from the north on 17 July 2015 (day 198), see Fig. 6. This northerly wind event had the expected impact on water temperature at the northeast (P2) and southwest (P3) platforms. At the peak of the northerly winds the thermocline upwelled at the northeast platform (decreasing temperature at shallow depths in Fig. 6c), and downwelled at the southwest platform (increasing temperature at depth in Fig. 6d). As the wind subsided during the night of 17 July 2015 (day 198), and then reversed to blowing from the south during the early morning of 18 July 2015 (day 199), the shallow water temperature rose at the northeast platform (P2, Fig. 6c), and the deeper water temperature fell at the southwest platform (P3, Fig. 6d).

The turbidity measured at a depth of 7.5 m at the southwest platform shows a large drop during the wind event (Fig. 6e). To understand why this occurs, recall that the 7.5 m sensor is located in a turbidity gradient that can be seen in the profile of 20 July 2015 (Fig. 4b). During the wind event, warm clear surface water displaces colder turbid water at 7.5 m at the downwind location of platform P3. Because of the strong vertical gradient in turbidity, vertical motions of the thermocline result in large rapid changes in turbidity.

This pattern repeats itself during every wind event throughout summer stratification (see Fig. 3). Note, that throughout the period from day 195–202, including during the strongest winds, the turbidity in the upper layer (red line) continues to decline (Fig. 5e). This occurs because the thermal stratification isolates the epilimnion from the potential source of higher turbidity water in the hypolimnion. The spectrum of the temperature, isotherms and turbidity (not shown) for this period match the spectrum of the wind with a peak between 0.3 and 0.5 cycles a day (period of 2–3 days). There are no peaks at the expected period of standing internal waves (shorter than 12 h) indicating these waves are probably damped.

#### Transition to fall turnover

**Fig. 7:**
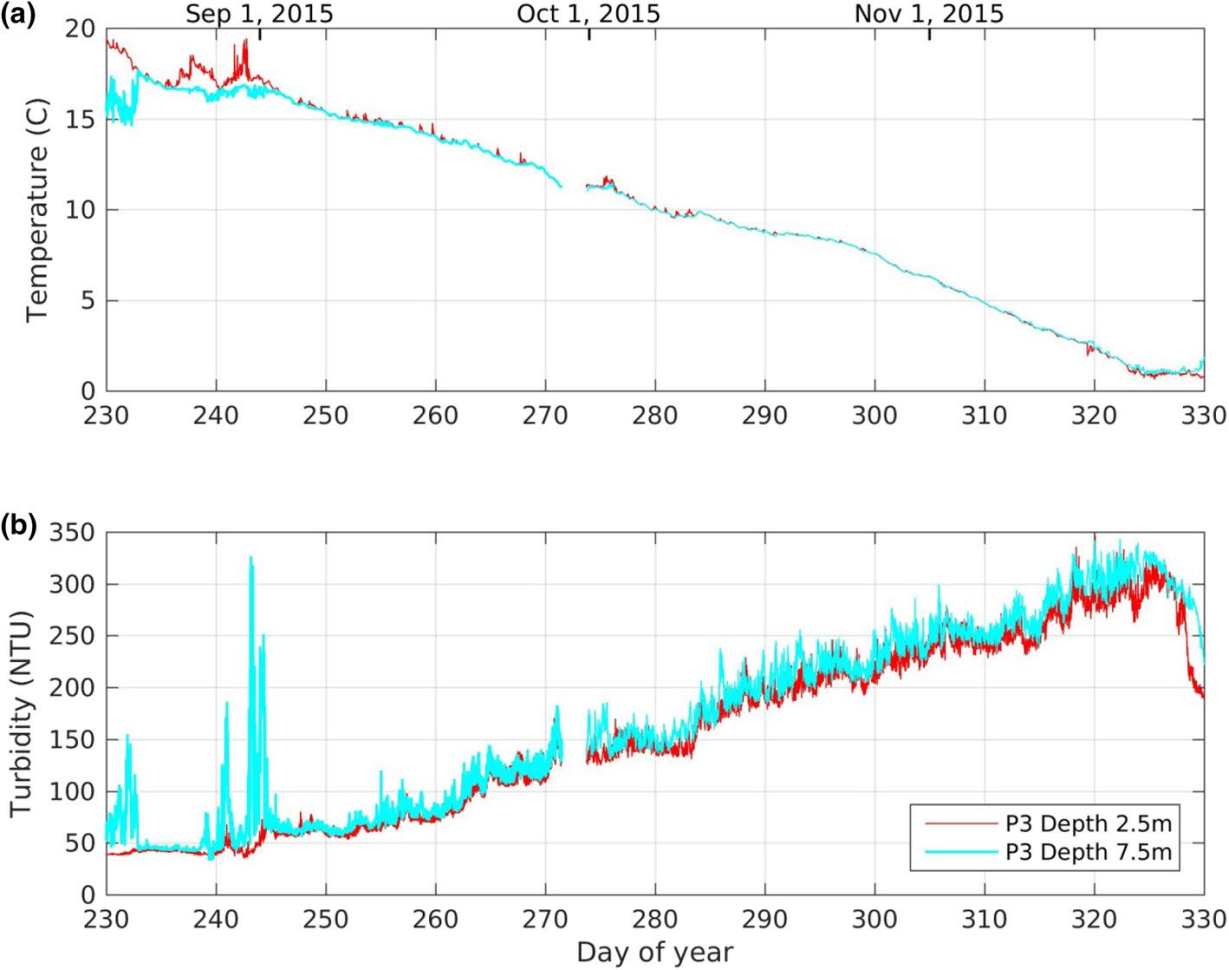
Temperature (**a**) and turbidity (**b**) measured at the southwest platform (P3) during fall turnover of 2015

In August, the epilimnion cools and deepens to the bottom of the water column (Fig. 3a). There follows a transition period of approximately 2 weeks, when, due to variations in wind and convective cooling, the water column intermittently re-stratifies. For example, in 2015 at the southwest platform, the water column was stratified until 20 Aug (day 232), was well mixed on Aug 21 and 22 (days 233 and 234), stratified again until Sep 3 (day 246), and then well mixed (except for periods less than a day) until after ice-on (Fig. 7a). During the period of intermittent mixing the lake may be well mixed at one location and stratified at another. Any stratification during this period is weak and consequently internal wave motions are potentially large resulting in rapid turbidity spikes, see Fig. 7b (days 230–233 and 238–244).

### Fall

At the onset of fall turnover (about day 246 in 2015) internal wave activity ceases and the turbidity at both 2.5 m and 7.5 m increase almost linearly until ice-on (Fig. 7). Field measurements at an adjacent site show that the increase in turbidity correlates closely with the rate at which energy is imparted to the water column [12, 22]. In fall the energy input is a combination of wind and convective cooling, which we estimate by applying the similarity scaling commonly used in the ocean (e.g. [13], and more recently in lakes (e.g. [21]:

6 $$ \upepsilon_{z} = \frac{{0.56u_{*}^{3} }}{kz} + 0.77J_{b} $$

where $\upepsilon_{z}$ is the rate of dissipation of turbulent kinetic energy; $u_{*}$ the shear velocity; $k = 0.4$, the von Karman coefficient;$z = 8\; {\text{m}}$, the mean water depth; and $J_{b}$, the surface buoyancy flux due to cooling. We use the procedures given in Imboden and Wüest [9] to calculate $u_{*}$ and $J_{b}$. The coefficients 0.56 and 0.77 were determined for a similarly sized lake during fall cooling [21]. Note that the buoyancy flux due to surface cooling drops to zero when the water temperature approaches the temperature of maximum density (~ 3.7 °C for BML), which occurs approximately a week before ice-on. This indicates that while convective cooling may be important, it is not the dominant mixing process, at least not in the latter part of fall.

Close inspection of Fig. 7b reveals that the increase in turbidity is not monotonic. There are several storm events, each lasting several days, when the average daily turbidity increases significantly, departing from the general upward trend. During the calm periods between these events, the turbidity typically decreases.

**Fig. 8:**
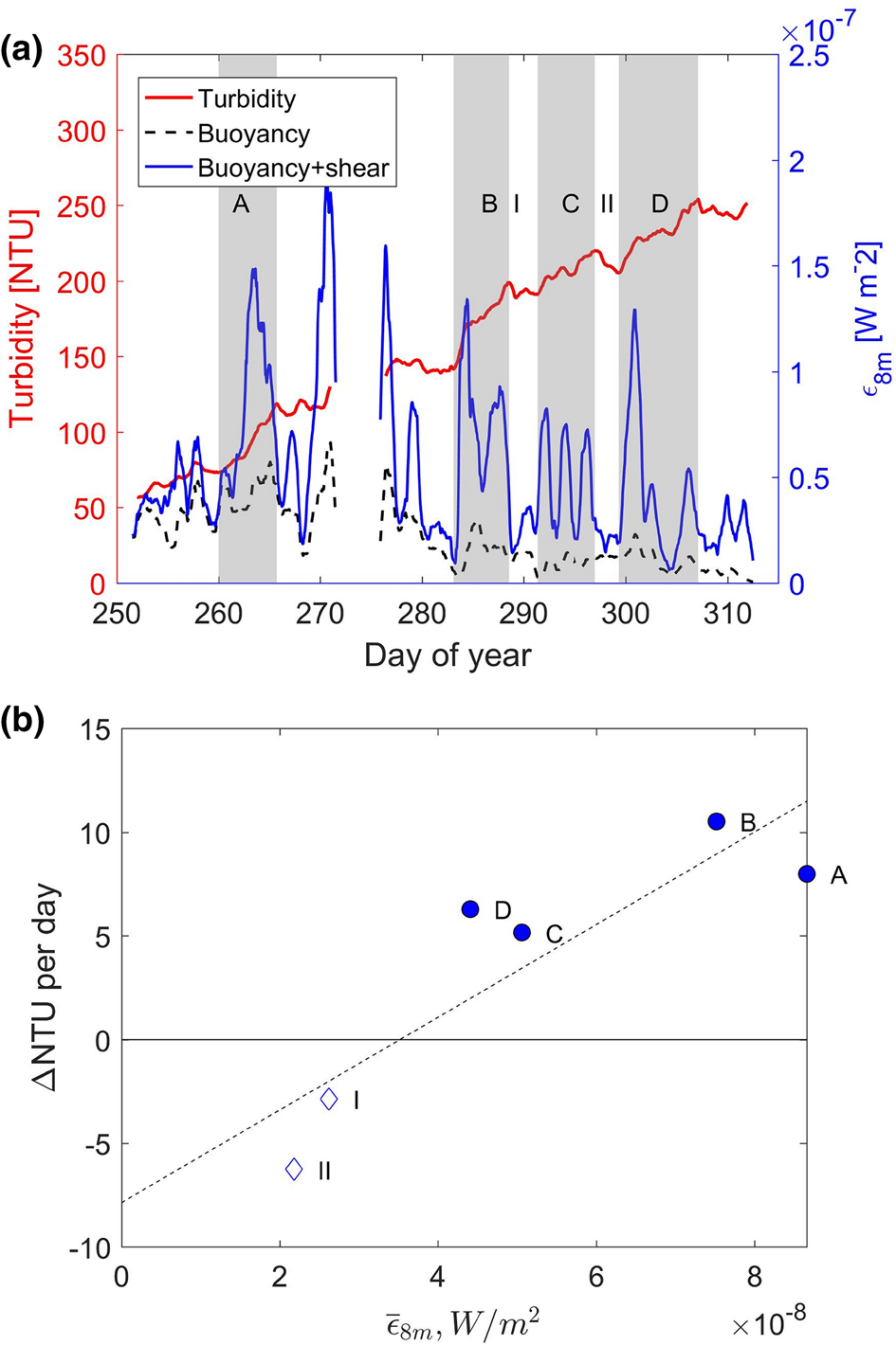
**a** Turbidity (red) and energy input from wind plus convection (solid blue line) calculated using similarity scaling (Eq. 6) and from convection only, i.e. 0.77J_b_ (dashed black line) at 2.5 m depth at the southwest platform (P3) from Sept. 9 (day 252) to Nov. 8 (day 312) 2015. Events (A–D) are periods during which the turbidity is generally increasing; periods between events are labelled I–II. **b** Energy input from wind and convection. Annual servicing of the instruments resulted in a data gap from day 272 to day 276

We compare the average daily increase in turbidity at 2.5 m depth at P3 during four storm events (A, B, C & D in Fig. 8a) between days 252 and 312 (Sept. 9 to Nov. 8) in 2015, with the input of turbulent kinetic energy into the water column due to wind and convective cooling in Fig. 8. At the beginning of fall cooling the contributions of the buoyancy flux and wind shear are of the same order of magnitude (Fig. 8a). As the water temperature approaches the temperature of maximum density the wind shear dominates since the buoyancy flux drops to zero. The total input of turbulent kinetic energy during the periods of rapid increase in turbidity is much greater than in the intervals between them (Fig. 8b). In general, the higher the energy input, the more rapid the observed rate of increase in turbidity. The rate of decrease in turbidity during periods of low energy input (periods I and II in Fig. 8a) is comparable with the decrease of turbidity in the epilimnion during summer shown in Fig. 5.

The general increase in turbidity, except during calm periods, continues until ice-on, at which time the turbidity decreases rapidly and the annual cycle as described above begins again.

### Interannual variability

Although all 4 years had the same general seasonal turbidity trends (decreasing and then steady in winter, increasing in the spring, decreasing in the summer, and increasing in the fall), there are several notable differences between the years in the turbidity levels and the timing of various events (Fig. 3 and Table 1).

1. First, although there is limited winter turbidity data, it was higher in both 2014 and 2015 than in 2016.
2. The lower winter 2016 turbidity levels continued after ice-off with spring maximums of 150 and 280 NTU at 2.5 m and 7.5 m, respectively, compared to 2015 spring maximums of 221 and 380 NTU at the same depths.
3. The minimum turbidity that occurred each summer in the epilimnion (i.e. 2.5 m depth), was 5, 10, 36, and 16 NTU in 2013, 2014, 2015 and 2016, respectively.
4. In terms of timing of events, 2016 showed the latest onset of summer stratification (June 23) compared to previous years of late May/early June. Fall turnover dates ranged from Aug. 27 to as late as Sept. 7. The timing of ice-on and ice-off was fairly consistent from year to year (see Table 1).

## Discussion

### Winter particle settling

**Fig. 9:**
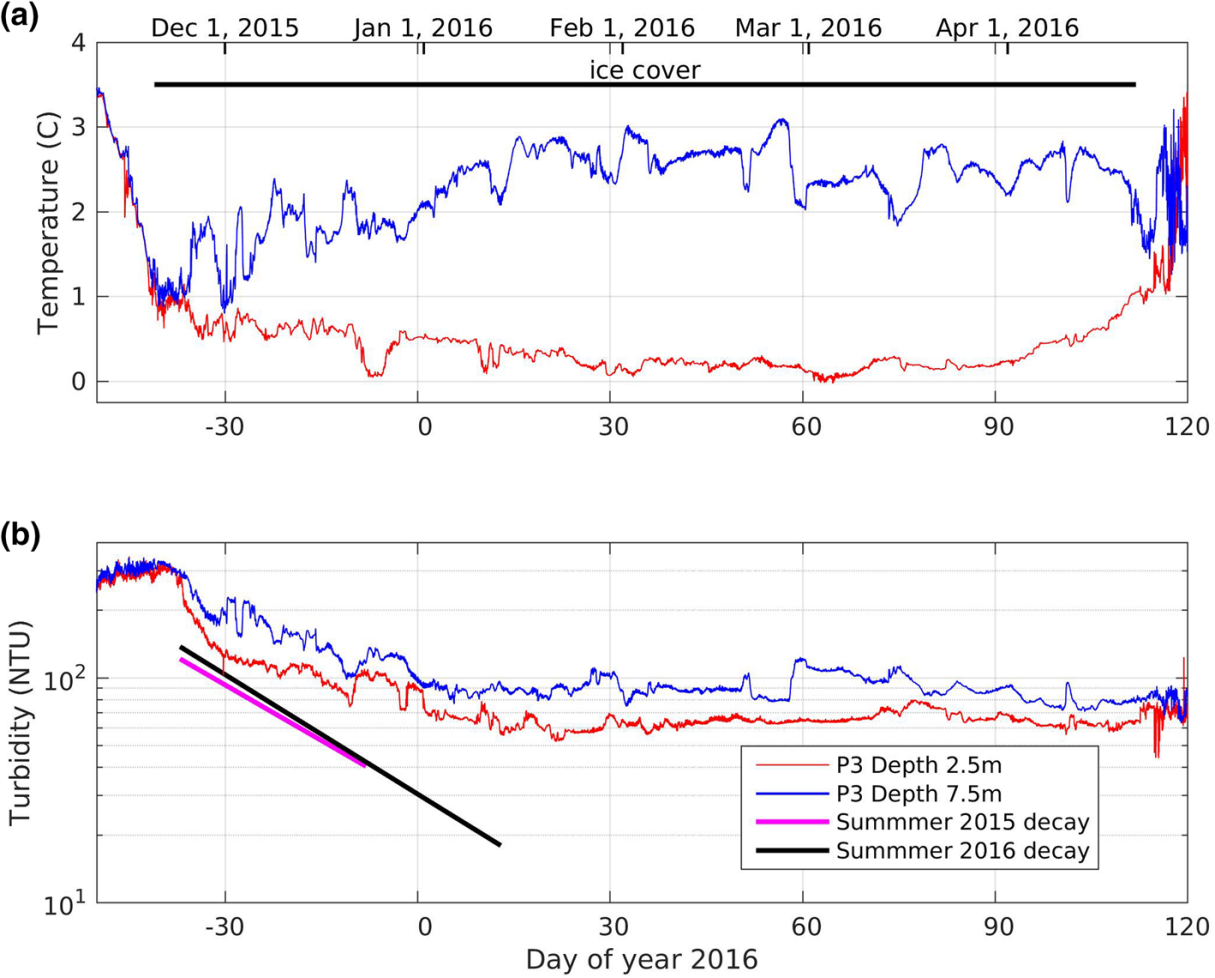
**a** Temperature and **b** turbidity measured at the southwest platform (P3) under ice. The fitted declines in turbidity in the epilimnion during the summers of 2015 and 2016 (see Fig. 5) are included in **b**. Note that the data plotted here is identical to the longer time series plotted in Fig. 3 and described in Sect. 4.1

Once ice formed on the lake in November 2015 turbidity initially decreased far more rapidly than during summer, see Fig. 9b. This decrease may be due to the rapid settling of relatively large particles that were remobilised into the water column during fall turnover. After the initial rapid decrease in turbidity at ice-on, the decay rate decreased until the second half of January 2016, after which time the turbidity remained relatively constant until ice-off. It is not clear why the turbidity levels off, or why the minimum turbidity in winter is much higher than the minimum turbidity in summer (Table 1).

One hypothesis for elevated winter turbidity may be gas ebullition from the FFT, and with bubbles rising through the water column (see [12], Fig. 4). It may be that the gas bubbles input enough energy into the water column to maintain a relatively high turbidity, or that they entrain some particles from the FFT (similar to the observations of [10]. Gas generation rates from methanogenesis in FFT depend on a number of factors including temperature. It is not known how the annual temperature variations (of up to 4 °C, [4] in the upper meter of the FFT, might impact ebullition rates or thermally driven convection. Another possible reason for elevated turbidity under the ice is salt water exclusion as the ice forms. Bluteau et al. [2] show that salt water exclusion can overcome the weak density stratification under the ice and facilitate mixing throughout the water column. The effects of gas ebullition and salt exclusion are the subject of ongoing field and laboratory investigations.

### Winter turbidity variations

**Fig. 10:**
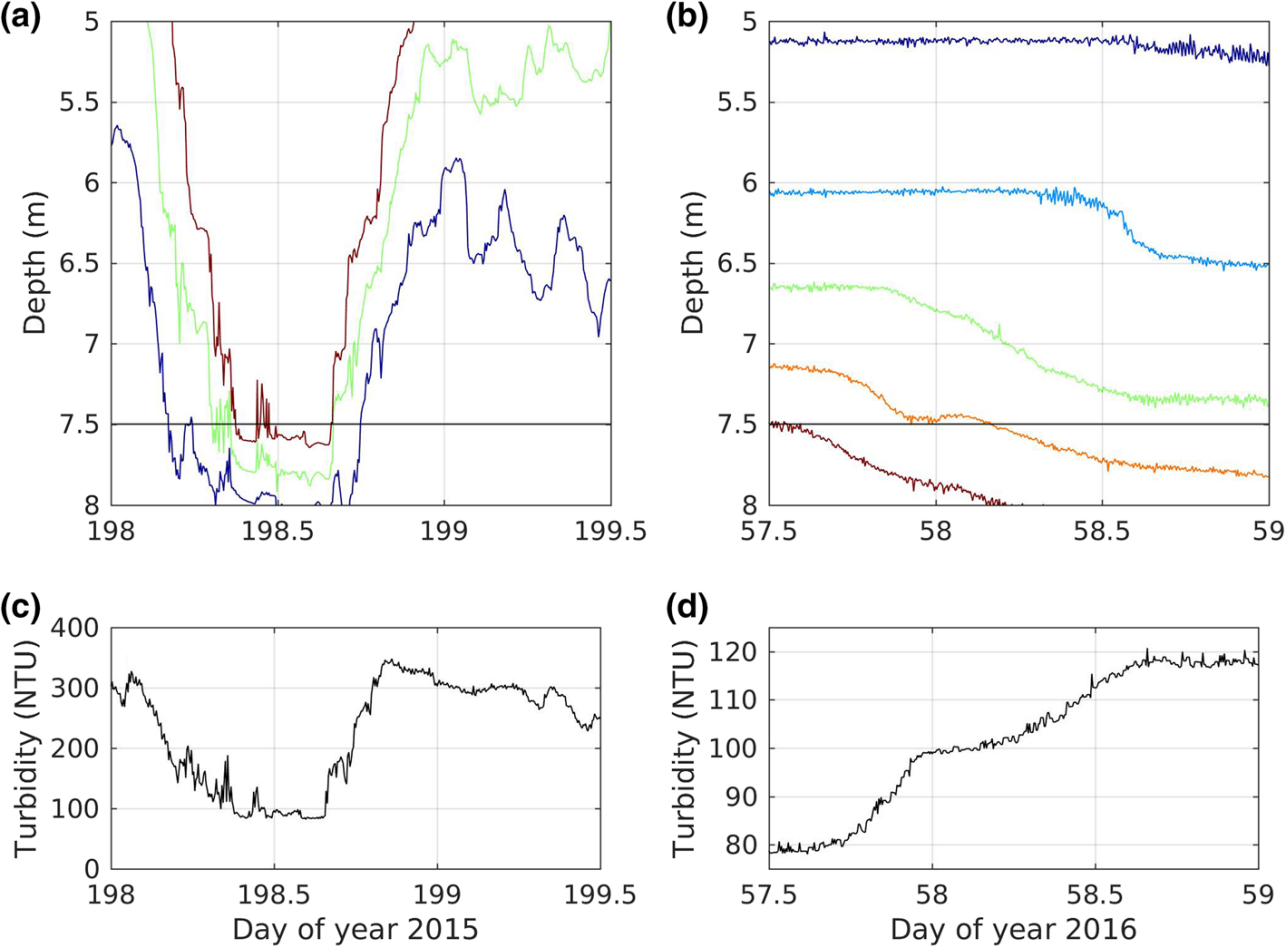
**a** Isotherms (14 °C, 16 °C, 18 °C) at the southwest platform (P3) during a summer seiche in 2015. **b** Isotherms (1 °C, 1.5 °C, 2 °C, 2.5 °C, 3 °C) at P3 during winter cooling in 2016. The horizontal black line in panels **a**, **b** indicates the depth of the turbidity sensor. **c**, **d** Turbidity at 7.5 m at P3. For clarity the plotted temperature and turbidity are sub-sampled at 5 min intervals

In addition to the decline in turbidity after ice-on, the turbidity exhibits variations at shorter time scales (< 10 days) throughout the winter. In Fig. 10 we compare the change in turbidity and isotherm depth that occurred over 1.5 days during the winter of 2016, with the change in turbidity and isotherm depth that occurred during the seiche event from the summer of 2015. Similar to the short time scale variations that occurred in the summer (Fig. 10a, c), the variations in turbidity were correlated with temperature (Fig. 10b, d). However, unlike in summer, variations in winter were not due to basin scale internal seiches, namely, in winter, the temperature at P2 (not shown) was not out of phase with the temperature at P3 (Fig. 3b).

Similarly, the largest variations in turbidity under the ice are not explained by downwelling of cooler, clearer water from above or by upwelling of warmer, more turbid water from depth. Note that downwelling appears as falling isotherms whether temperature is decreasing with depth (summer, Fig. 10a) or increasing with depth (winter, Fig. 10b). For example, on days 57 and 58, during the increase in turbidity which suggested upwelling (Fig. 10d), the temperature isotherms were falling suggesting downwelling (Fig. 10b). These changes suggest instead, that water masses move horizontally rather than vertically past the turbidity sensor, consistent with gradients in turbidity and temperature observed across the basin in the winter. The connection between atmospheric forcing and changes in water temperature and turbidity under the ice is the subject of ongoing study at Base Mine Lake.

### Spring turbidity

While both wind shear and convective cooling contribute to mixing throughout most of the fall, for most of the spring, surface heating stabilises the water column and inhibits mixing. In addition, in the spring the water column may be stabilised by fresh water ice melt. As a consequence, while in the fall the water column is almost always well mixed; in spring the water column is only intermittently well mixed (Fig. 3a). In some circumstances spring turnover can be suppressed altogether [16]. Consequently, the increase in turbidity in spring is far more variable than in fall, and the formulation presented in Eq. 6 is not applicable.

## Conclusions

We investigated primary processes that cause seasonal variability in mineral turbidity in Base Mine Lake. The annual turbidity cycle is summarized as follows: during summer there is an exponential decrease in turbidity due to relatively steady settling of particulate matter from the epilimnion, through the thermocline to the base of the water column. Rapid changes (shorter than 1 day) in turbidity during summer are due to wind-driven internal seiches. Convective cooling and wind during fall storm events increase the turbidity to an annual maximum just before ice-on. Ice-on suppresses wind forcing, resulting in a rapid decrease in turbidity. By mid-January the turbidity falls to a relatively constant value that is higher than the summer minimum. The reason(s) for this relatively high minimum turbidity in winter is, as yet, unexplained; however, we note that it may be sustained convection driven by salt exclusion as ice thickens, heating from warm underlying FFT, or by ebullition from the FFT. Following ice-melt, wind increases turbidity until summer stratification begins and turbidity declines.

## References

[1] Bloesch J, O’Sullivan PE, Reynolds CS (2004). Sedimentation and lake sediment formation. The lakes handbook—limnology and limnetic ecology.

[2] Bluteau CE, Pieters R, Lawrence GA (2017). The effects of salt exclusion during ice formation on circulation in lakes. Environ Fluid Mech.

[3] Cózar A, Gálvez JA, Hull V, García CM, Loiselle SA (2005). Sediment resuspension by wind in a shallow lake of Esteros del Ibera (Argentina): a model based on turbidimetry. Ecol Model.

[4] Dompierre KAH (2016) Controls on mass and thermal loading to an oil sands end pit lake from underlying fluid fine tailings. Unpublished Ph.D. thesis, Department of Civil, Geological, and Environmental Engineering, University of Saskatoon, Saskatoon, Saskatchewan, Canada, vol 141, pp, 24–44

[5] Dompierre KA, Lee Barbour S, North RL, Carey SK, Lindsay MB (2017). Chemical mass transport between fluid fine tailings and the overlying water cover of an oil sands end pit lake. Water Resour Res.

[6] Dubourg P, North RL, Hunter K, Vandergucht DM, Abirhire O, Silsbe GM, Guildford SJ, Hudson JJ (2015). Light and nutrient co-limitation of phytoplankton communities in a large reservoir: Lake Diefenbaker, Saskatchewan, Canada. J Great Lakes Res.

[7] Heiskanen JJ, Mammarella I, Ojala A, Stepanenko V, Erkkilä K-M, Miettinen H, Sandström H, Eugster W, Leppäranta M, Järvinen H, Vesala T, Nordbo A (2015). Effects of water clarity on lake stratification and lake-atmosphere heat exchange. J Geophys Res Atmos.

[8] Hocking GC, Straškraba M (1999). The effect of light extinction on thermal stratification in reservoirs and lakes. Int Rev Hydrobiol.

[9] Imboden DM, Wüest A, Lerman A, Imboden DM, Gat JR (1995). Mixing mechanisms in lakes. Physics and chemistry of lakes.

[10] Kavcar C (2008) Stability of cohesive sediments subject to pore water and gas ebullition fluxes and effectiveness of sand and AquaBlok Caps in reducing the resuspension rates. Unpublished Ph.D. thesis, University of Michigan, vol 206, pp. 89–94

[11] Lawrence GA, Ward PRB, McKinnon MD (1991). Wind-wave induced suspension of mine tailings in disposal ponds. Can J Civ Eng.

[12] Lawrence GA, Tedford EW, Pieters R (2016). Suspended solids in an end pit lake: potential mixing mechanisms. Can J Civ Eng.

[13] Lombardo CP, Gregg MC (1989). Similarity scaling of viscous and thermal dissipation in a convecting surface boundary layer. J Geophys Res Oceans.

[14] Haneef-Mian M, Yanful EK (2007). Erosion characteristics and resuspension of sub-aqueous mine tailings. J Environ Eng Sci.

[15] McNally W, Friedrichs C, Hamilton D, Hayter E, Shrestha P, Rodriguez H, Sheremet A, Teeter A, ASCE Task Committee on Management of Fluid Mud (2007). Management of fluid mud in estuaries, bays and lakes. I: present state of understanding on character and behaviour. J Hydraul En.

[16] Pieters R, Lawrence GA (2009). Effect of salt exclusion from lake ice on seasonal circulation. Limnol Oceanogr.

[17] Reynolds CS (1984). Phytoplankton periodicity: the interactions of form, function and environmental variability. Freshw Biol.

[18] Rose KC, Hamilton DP, Williamson CE, McBride CG, Fischer JM, Olson MH, Saros JE, Allan MG, Cabrol N (2014). Light attenuation characteristics of glacially fed lakes. J Geophys Res Biogeosci.

[19] Smith DG, Croker GF, McFarlane K (1995). Human perception of water appearance. NZ J Mar Freshw Res.

[20] Smith IR (1982). A simple theory of algal deposition. Freshw Biol.

[21] Tedford EW, MacIntyre S, Miller SD, Czikowsky MJ (2014). Similarity scaling of turbulence in a temperate lake during fall cooling. J Geophys Res Oceans.

[22] Ward PRB, Lawrence GA, MacKinnon MD (1994) Wind-driven re-suspension of sediment in a large tailings pond. In: Proceedings, international symposium on ecology and engineering, Oct 29 to Nov 3, 1994, vol VI. Organized by Coastal and Offshore Engineering Institute, Universiti Teknologi Malaysia, and Centre for Water Research, University of Western Australia, pp 37–57

[23] Wetzel RG (2001). Limnology: lake and river ecosystems.

[24] Wüest A, Zeh M, Ackerman JD (2007). Lake Brienz project: an interdisciplinary catchment-to-lake study. Aquat Sci.

